# Bronchoalveolar lavage in infants with recurrent lower respiratory symptoms

**DOI:** 10.1186/2045-7022-4-35

**Published:** 2014-10-24

**Authors:** Kristiina Malmström, Maili Lehto, Marja-Leena Majuri, Timo Paavonen, Seppo Sarna, Anna S Pelkonen, L Pekka Malmberg, Harry Lindahl, Merja Kajosaari, Sejal Saglani, Harri Alenius, Mika J Mäkelä

**Affiliations:** Department of Allergy, Helsinki University Central Hospital, PO Box 160, Helsinki, FI 00029 Finland; Institute of Occupational Health, Helsinki, Finland; Department of Pathology, Fimlab Laboratories, Tampere University Central Hospital, and University of Tampere, Tampere, Finland; Department of Public Health, University of Helsinki, Helsinki, Finland; Hospital for Children and Adolescents, Helsinki University Central Hospital, Helsinki, Finland; Department of Respiratory Pediatrics, Imperial College London, London, UK

**Keywords:** Bronchoalveolar lavage, Infant, Pro-inflammatory cytokines, Pulmonary function, Wheeze

## Abstract

**Background:**

Few data are available about the inflammatory cytokine profile of bronchoalveolar lavage (BAL) from young children with frequent wheeze. The first aim was to investigate the BAL cellular and cytokine profiles in infants with recurrent lower respiratory symptoms in whom bronchoscopy was indicated for clinical symptom evaluation. The second aim was to relate the BAL results with the histological findings of the endobronchial carina biopsies.

**Methods:**

Thirty-nine infants (median age 0.9 years) underwent lung function testing by whole-body plethysmography prior to the bronchoscopy. The BAL differential cell counts and cytokine levels were quantified. These findings were compared with the histological findings of the endobronchial carina biopsies.

**Results:**

The differential cytology reflected mainly that described for healthy infants with lymphocyte counts at the upper range level. A positive association between BAL CD8+ lymphocytes and neutrophils and endobronchial reticular basement membrane was found. Detectable levels of pro-inflammatory cytokine proteins IL-1β, IL-17A, IL-18, IL-23, and IL-33 were found, whereas levels of Th2-type cytokine proteins were low. Frequent wheeze was the only clinical characteristic significantly related to detectable combined pro-inflammatory cytokine profile. Lung function did not correlate with any cytokine.

**Conclusions:**

A positive association between BAL CD8+ lymphocytes and neutrophils and endobronchial reticular basement thickness was found. Detectable production of pro-inflammatory cytokines associated positively with frequent wheeze.

## Introduction

Two characteristic pathological features of asthma, thickening of the reticular basement membrane and eosinophilic airway inflammation, are absent in symptomatic infants with reversible airflow obstruction [1] but already present in preschool children with severe, recurrent wheeze [2]. Instead, a network of antigen presenting dendritic cells and macrophages, bronchus associated lymphoid tissue structures, and Foxp3+ regulatory T cells have been detected in endobronchial biopsies already in these symptomatic infants [3].

Bronchoalveolar lavage (BAL) is a standardized method to obtain cells from the airway lumen [4]. Increased numbers of eosinophils and mast cells have been detected in BAL from school-aged children with atopic asthma but not from preschoolers with viral wheeze [5]. In another study, increased numbers of neutrophils and epithelial cells were detected in BAL samples from school-aged asthmatics and from young preschool children with viral-induced wheeze [6]. Recently, children (5–17 years) with severe therapy-resistant asthma had significantly increased BAL eosinophils compared control subjects [7] but no increase in BAL fluid interleukin (IL)-4, IL-5, or IL-13 levels. Interestingly a subgroup of children with Th2 cytokines had significantly lower lung function than those with undetectable BAL fluid Th2 cytokines. To our knowledge, there is no data about the inflammatory cytokine profile of BAL samples from children less than two years of age with troublesome lower airway symptoms and wheeze.

The present study afforded us a unique opportunity to investigate the BAL cellular and cytokine profiles in a group of infants with defined lung function [1] in whom bronchoscopy was indicated for clinical symptom evaluation. Secondly, the BAL findings were compared with the histological findings of the endobronchial carina biopsies.

## Methods

### Subjects

The study was established in January 29, 2000 and ended in June 18, 2003. The children were referred to a tertiary center for investigation of recurrent lower respiratory tract symptoms (including dyspnea, cough, and wheeze). As a part of their clinical assessment they underwent lung function testing by whole body plethysmography at the mean age of 0.9 years followed by bronchoscopy, BAL and bronchial biopsy to exclude structural airway abnormalities, such as subglottic stenosis, laryngo-, tracheo-, or bronchomalacia, and other diagnosis, such as foreign body inhalation or mucus plugging [1]. The patients were excluded if they had used corticosteroids within 8 weeks of their lung function visit. Atopy was defined by a positive skin prick test to food or aeroallergens described earlier [1]. Frequent wheeze was defined as ≥3 separate doctor confirmed wheezing episodes. Functional residual capacity, specific airway conductance, and bronchodilator responsiveness were measured (+ve if specific airway conductance increased >30%) [1]. Abnormal baseline lung function was considered when specific airway conductance was lower than that of 5^th^ percentile (Z-score < -1.65) of the reference range [8]. The clinical diagnose was based on history, lung function, bronchoscopy, and follow-up at 3 years of age and the children were categorized having “asthma”, “structural abnormality”, or “unspecified diagnosis”.

### Bronchoscopy, BAL and endobronchial biopsy

It had been routine clinical practice at the Hospital for Children and Adolescents and Skin and Allergy Hospital, University of Helsinki, to evaluate infants with either chronic or recurrent respiratory difficulties using rigid bronchoscopy. A pediatric surgeon performed bronchoscopy and endobronchial biopsy under general anesthesia, with a size 3.5 Stortz rigid bronchoscope, having an outer diameter of 5.7 mm. BAL was performed with Pentax FI-10BS bronchofiberoscope with 3.5 mm outer diameter. The fiberoscope was wedged into a segmental bronchus of the right lower lobe and 4×5 ml aliquots of sterile saline were instilled and suctioned back on ice. Since the first suctions will include some bronchial mucus and epithelium, two first and two last suctions were combined resulting in two separate fractions whereas the second fraction would represent best the alveolar level.

The carina biopsies taken from the main carina were processed and analyzed for the thickness of reticular basement membrane by light microscopy and for subepithelial inflammatory cell counts (eosinophils, neutrophils, mast cells, plasma cells, lymphomononuclear cells) indentified by their ultrastructure using transmission electron microscopy as described earlier [1].

### Cytokine analysis of BAL samples

The volume of retrieved BAL fluid was measured, cells were counted and cell suspension was cytocentrifuged onto microscopic slides. Epithelial and red blood cells, differential cell counts, and CD4/CD8-lymphocytes were determined [4].

Extraction of cellular RNA from BAL cells, synthesis of cDNA and quantitative Real Time-PCR were performed for IL-10, IL-13, IL-17A, IL-18, IL-33, TGF-1β, TSLP (thymic stromal derived lymphoprotein), Foxp3, ST2, and IL-1RAcP [9]. The house-keeping gene, ribosomal 18S, was used during RT-PCR for normalization. Cytokine protein analysis from BAL supernatants for IL-1β, IL-4, IL-9, IL-12p70, and INF-α2 were made with the Luminex bead system (Bio-Plex 200 System) by labeled cytokine capture antibody pairs (Bio-Rad Laboratories), whereas other cytokines were measured with commercial ELISA kits: IL-10, IL-17A, and IL-23 (eBioscience, Inc., San Diego, CA, USA), IL-13 (Eli-pair, Diaclone, Besancor Cedex, France), IL-18 (Bender MedSystems, Vienna, Austria), IL-28, TGF-β1, and TSLP (R&D Systems, Minneapolis, MN, USA), IL-33 (Apotech, Epalinges, Switzerland).

### Statistical analysis

Mann–Whitney U-test was used for comparisons of BAL findings between the infant groups. Associations between clinical parameters and BAL findings were calculated with Chi-Square and Fisher’s tests. Correlations between cellular and cytokine findings were calculated with Spearman’s correlation-test. Two-sided p-values <0.05 were considered statistically significant. The Ethics Committee of Diseases of Children and Adolescents at the Helsinki University Central Hospital approved the study protocol and written informed parental consents were obtained.

## Results

BAL samples were obtained from 39 infants: 30 children with abnormal lung function and 9 children with normal lung function. Asthma was diagnosed in 34, structural abnormalities in 2 and prolonged mucus or cough in 3 children. In addition, mild structural abnormalities were detected in 3 children with asthma. Structural abnormalities included 2 laryngo-,1 tracheo- and 1 bronchomalacia and 1 child had enlarged adenoidal tissue. A description of the children is presented in the Table 1.

**Table 1:** **Characteristics of infants at the time of lung function test and findings of bronchial biopsy**

N	39
Age, years, median (range)	0.88 (0.28–1.94)
Sex, male	25 (64%)
Parental asthma	26 (41%)
Parental smoking	9 (23%)
Symptoms	
Age at first symptoms (y)	0.25 (0–1.33)
Duration (y)	0.58 (0.17–1.33)
Wheeze	23 (59%)
Frequent wheeze*	15 (38%)
Cough	26 (67%)
Mucus	23 (59%)
Dyspnea	5 (13%)
SPT+	15 (38%)
SPT + &/or eczema	24 (62%)
Lung function	
Abnormal	30 (77%)
Abnormal with + ve BDR	12 (40%)
sGaw, z-score	-2.4 (-5.2–0.9)
FRC, z-score	0.37 (-1.5–1.9)
Bronchial biopsy findings	
RBM, μm	3.8 (2.7–6–0)
Plasma cells/area	1.3 (0–13.8)
Neutrophils/area	0.07 (0–0.88)
Mast cells/area	0.39 (0–1.26)
Lymphocytes/area	1.7 (0–16.9)
Total cells/area	31.4 (16.9–138.9)
Dg at infancy	
Asthma	34 (87%)
Structural abnormality	5 (13%)
Unspecified	3 (8%)

Lung function and bronchial biopsy findings are presented as medians (range).

*Frequent wheeze, ≥ 3 times.

SPT, skin prick test; BDR, bronchodilator response (sGaw >30%); sGaw, specific airway conductance; FRC, functional residual capacity; RBM, reticular basement membrane.

### BAL cell profiles and correlations with endobronchial cell counts and RBM

BAL cellular findings are presented in Table 2. Macrophages were predominant cells in both fractions followed by lymphocytes, whereas eosinophils were absent. The number of lymphocytes in endobronchial biopsy correlated positively with BAL fraction 1 CD8-lymphocytes (r = 0.734, p = 0.007; Spearman) and negatively with ratio of CD4/CD8-lymphocytes (r = -0.739, p = 0.037; Spearman). The thickness of RBM correlated positively with the BAL fraction 1 CD8+ lymphocyte % (r = 0.488, p = 0.047; Spearman), and with BAL fraction 2 lymphocyte % (r = 0.473, p = 0.017; Spearman) and neutrophil % (r = 0.499, p = 0.011; Spearman). There were no differences in BAL cellular findings between the children with and without infant asthma, neither between the children with normal and abnormal lung function.

**Table 2:** **Bronchoalveolar cell profiles**

	BAL fraction 1	BAL fraction 2
N	39	34
Median of BAL fluid recovery volume (ml)	2.0 (0.3–5.4)	3.2 (0–6.8)
Median of BAL fluid recovery volume (%)	20 (3–54)	32 (0–68)
Median of total cell count (× 10^4^/ml)	0.0 (0–29.7)	1.9 (0–15.5)
Median of differential BAL non-epithelial cell count		
Macrophages (%)	72.0 (0–95)	82.0 (0–98)
Lymphocytes (%)	6.0 (0–19)	13.0 (0–49)
Neutrophils (%)	5.0 (0–82)	2.0 (0–35)
Eosinophils (%)	0.0 (0–2)	0.0 (0–1)
Basophils (%)	0.0 (0–2)	0.0 (0–2)
CD4+ (%)	10 (0–50)	19.5 (0–65)
CD8+ (%)	18.0 (0–78)	39.0 (0–70)
CD4+/CD8+	0.63 (0–2.14)	0.43 (0–2.17)
Median of epithelial cell count (%)	15.0 (0–100)	5.0 (0–80)

*The results are expressed as median (range).

### BAL cytokines

**Figure 1:**
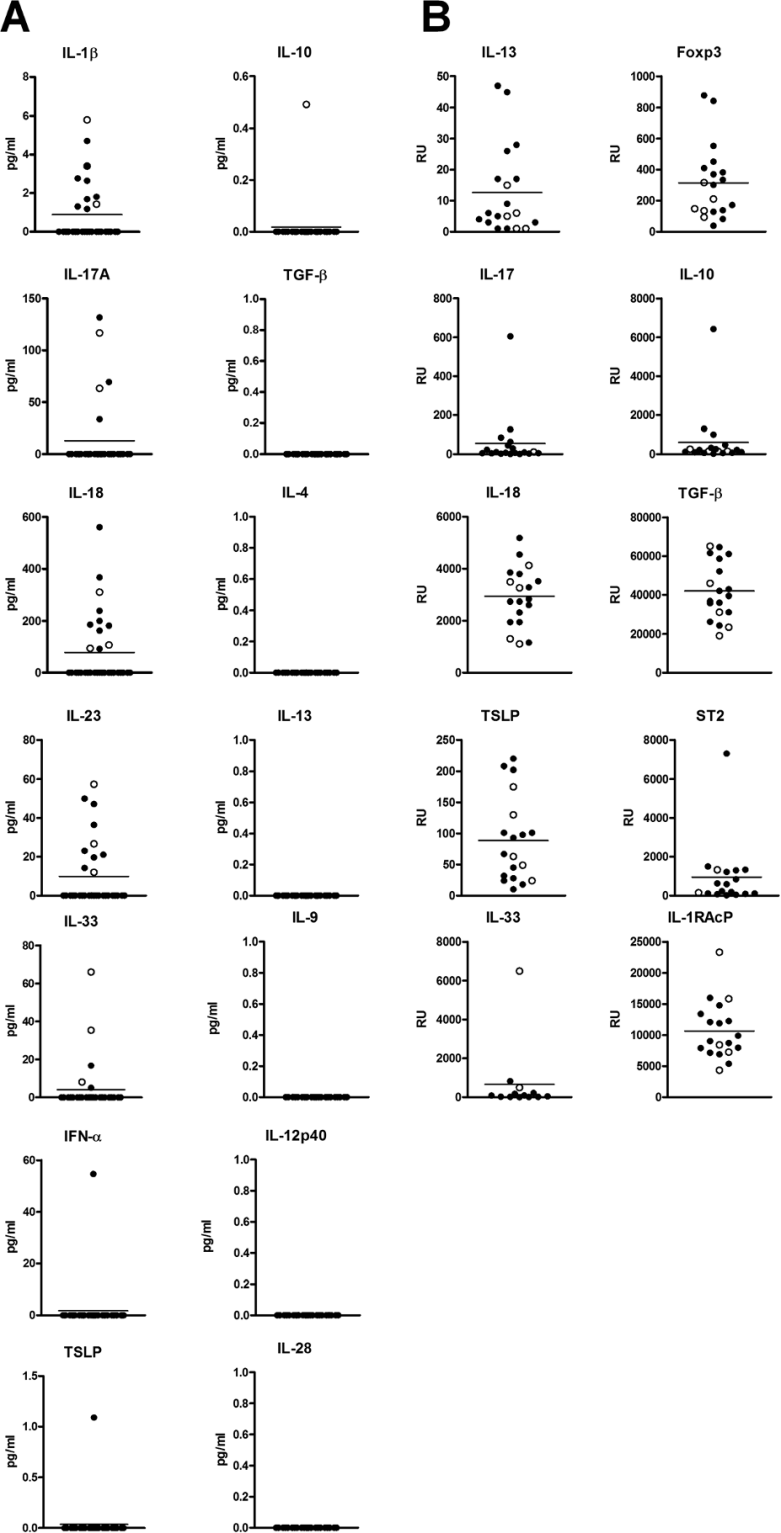
**Bronchoalevolar lavage fluid cytokine proteins (A) and mRNA expression of bronchoaleveolar lavage cell cytokines (B).** White circles, children with normal lung function. Black circles, children with abnormal lung function. Lines are means of all data. RU, relative units.

Analyses of cytokine proteins in BAL fluid and of cytokine mRNAs in BAL cells were available from 32 and 36 patients. Detectable levels of pro-inflammatory cytokine proteins IL-1β, IL-17A, IL-18, IL-23, and IL-33 were found whereas levels of Th2-type cytokine proteins, such as IL-4, IL-9, IL-13, and TSLP, were undetectable (Figure 1A). Besides, 13 children had low BAL cytokines for all measured cytokines. At the mRNA level, the expression of many cytokines were observed i.e. pro-inflammatory cytokine IL-18, TGF-β1, IL-13 and TSLP. In addition, the mRNA expression of regulatory T-cell marker Foxp3^+^ as well as RacP, co-receptor IL-1 for IL-1β, IL-33 and IL-36 were discovered (Figure 1B). Again, there were no differences in cytokine proteins in BAL fluid or cytokine mRNA expression between the children with and without infant asthma, neither between the children with normal and abnormal lung function.

### BAL cytokine correlations

**Figure 2:**
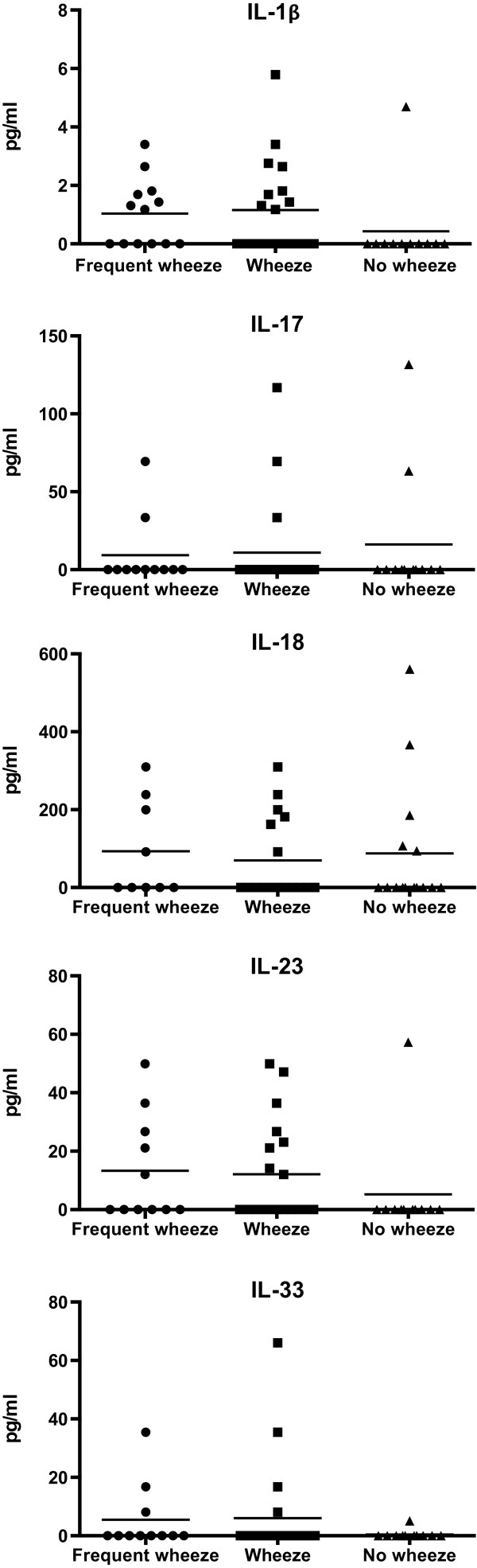
**Pro-inflammatory cytokine protein levels of bronchoalveolar lavage supernatants in frequent wheezers, wheezers and non-wheezers.** Frequent wheeze and wheeze were significantly (p = 0.004 and p = 0.029, Fisher) related to the detectable combined pro-inflammatory cytokine profile (IL-1β, IL-17, IL-18, IL-23, and IL-33). Lines express means of data.

The group of 19/32 (59%) patients with detectable levels of pro-inflammatory cytokine protein production (IL-1β, IL-17A, IL-18, IL-23, and IL-33) was evaluated for clinical characteristics. Frequent wheeze and wheeze were the only clinical characteristics significantly related to this detectable combined pro-inflammatory cytokine profile (p = 0.004 and p = 0.029, Fisher) (Figure 2). Neither lung function, atopy, duration of symptoms, age at the beginning of the symptoms, endobronchial cell counts, nor thickness of reticular basement membrane showed any associations with the pro-inflammatory cytokines. Detectable level of IL-1β was positively correlated with BAL fraction 1 lymphocytes and CD4+ lymphocytes (r = 0.526, p = 0.014; r = 0.537, p = 0.039; Spearman). In addition, detectable level of IL-17A was positively correlated with BAL fraction 2 macrophages (r = 0.438, p = 0.032; Spearman).

## Discussion

The present study characterized BAL cell counts and cytokine profiles in a group of infants with recurrent lower respiratory tract symptoms of whom 87% were diagnosed for asthma. For ethical reasons a healthy control group could not be included. Macrophages were predominant cells followed by lymphocytes. In addition, detectable levels of pro-inflammatory cytokine proteins IL-1β, IL-17A, IL-18, IL-23, and IL-33 were found.

The role of bronchial epithelial eosinophilia has been controversial in childhood asthma [1, 2, 10, 11]. Bronchial epithelial eosinophilia was not detected in the bronchial biopsies from the same corticosteroid-naive infants than in the present study [1]. The absence of BAL fluid eosinophilia in the present study supports the observations [6] that other cells than eosinophils are important in symptomatic children at the early age.

We found a positive association between the percentage of BAL fraction 1 CD8+ lymphocytes and thickness of reticular basement membrane and the number of lymphocytes in the endobronchial biopsy. In addition, a positive association was found between the percentages of BAL fraction 2 lymphocytes and neutrophils and the thickness of reticular basement membrane. These findings may have important implications since CD8+ lymphocytes potentiate development of airway hyperresponsiveness in experimental models and may amplify inappropriate immune responses seen in developing asthma [12]. Van Rensen and co-workers investigated the prognostic significance of bronchial CD8+ lymphocytes, eosinophils, and reticular basement membrane thickness for the subsequent decline in lung function in adult asthmatics after 7.5 years of follow-up [13]. They found an annual decline of lung function that correlated with bronchial CD8+ lymphocytes but not with eosinophils or reticular basement membrane thickness. They speculated that since CD8+ lymphocytes can induce potential conditions that are required for changes in airway structure, this may eventually lead to changes in airway structures.

Activation of pro-inflammatory cytokine production is a requirement for the adaptive immune system function. In this study, detectable levels of pro-inflammatory cytokines were found in BAL samples especially among infants with frequent wheeze regardless of current lung function. We did not find Th2 dominance (IL-4, IL-13, IL-9) or differences in the regulatory cytokines (i.e. IL-10, TGF-β1). In accordance to our findings, a significant T-cell driven airway inflammation was absent in mild/non-atopic 2–12 year old asymptomatic children with episodic wheeze according to intracellular cytokine IFN-γ, IL-2, IL-4, IL-5, and IL-10 analyses of BAL fluid [14]. Absence of Th2 cytokines IL4, IL-5 and IL-13 was also detected in school children (median age 12 years) with severe treatment-resistant asthma regardless of bronchial eosinophilia [7]. Pro-inflammatory cytokine IL-18 is constitutively expressed in a wide range of cells, including macrophages, dendritic cells, epithelial cells, and endothelial cells [15]. Thus, it is possible that IL-18 protein in BAL fluids originates from different lung tissue cells for instance from damaged airway epithelial cells as well as from macrophages circulating in BAL fluids. Production of pro-inflammatory cytokines is assumingly part of normal immunological maturation process during infancy. It is likely that children with frequent wheeze suffer from repeated viral infections, which may upgrade production of pro-inflammatory cytokines.

The instilled saline volume varies in protocols but 3 ml/kg has been recommended [4]. A smaller instilled volume in the present study, 20 ml/infant averaging 2 ml/kg, could explain low recovery volume and total cell number. The differential cytology reflected mainly that described for healthy infants in another studies recognizing that normal value range is wide and overlapping [4, 16]. Eosinophils were absent whereas other inflammatory cells were found. Median lymphocyte count 13% in fraction 2 was at the upper scale of that described for very young healthy controls [4] and wheezing infants [6].

We conclude that a positive association between percentage of BAL CD8+ lymphocytes and neutrophils and the thickness of reticular basement membrane in the endobronchial biopsy was found. Pro-inflammatory cytokines were detected in BAL fluids of infants with recurrent respiratory symptoms regardless of current lung function. Frequent wheeze and wheeze were the only characteristics significantly associated with detectable levels of pro-inflammatory cytokines.

## Abbreviations

BAL: Bronchoaleveolar lavage
IL: Interleukin
TSLP: Thymic stromal derived lymphoprotein.
